# Editorial

**Published:** 1874-09

**Authors:** 


### Editorial.

Some cynic has said "the world has been trying for sixty centuries to pluck the mote from its brother's eye, and never yet detected the beam in its own." Hence, while the suggestion conveyed in the aphorism is by no means novel, its practical application might prove a refreshing variety to the monotony of life in these dog-days. We shall propose very gravely, then, that we of the medical profession begin to extract the beam from our own eyes as indicated to us by the general public, on several recent occasions, through its exponent, the secular press.

Twice, of late, have charges been made against our profession, seriously compromising its honor and trustworthiness, if true. That they might have been made once, vaguely and generally, without foundation and unjustly, is probable; but when they are reiterated distinctly and specifically without contradiction, they thereby acquire a degree of importance which demands our attention.

The medical profession of the city of Chicago has been charged with collusion with druggists to rob the people—their patients—whose lives have been placed confidentially in their hands.

The general charge has been specified as follows:

1. In advertising certain pharmacists by circulating their business cards in the form of prescription papers, supplied at the expense of the pharmacist, which expenditure must be compensated for by the addition of a percentage to the reasonable profit received by the pharmacist upon his prescription trade.

2. In occupying offices, leased from pharmacists, without, or with a nominal, rental, the sums thus disbursed for such rents being "made up" in the manner already indicated, *i. e.*, by an additional charge to patients for medicines.

3. In receiving from pharmacists an actual percentage, in money, upon the amount charged for every prescription written by the physician and prepared by the pharmacist.

Are these charges true? If so, our assertion that they seriously compromise the honor and dignity of our profession is just; for their truth demonstrates the fact that certain members of our profession have become mere stipendiaries of certain pharmacists—"drummers" for the drug trade, and not even first-class drummers, salaried employees, but, like the rank and file of the numerous army of "commercial travelers," "working on commission."

We have made diligent inquiry to ascertain if any evidence of such collusion as is charged existed, and must say, "*Si non e vero e ben trovato.*"

That the practices specified are common, is well known. They have been sanctioned by custom, and adopted by a large number of physicians, upon whom no suspicion of dishonor could for a moment rest. But that they are applied dishonestly by some, there is good reason to believe. That these usages or customs, innocent, perhaps, in their inception, are susceptible of outrageous abuse, must be clear to any honest mind; and while there are many who still adhere to them in perfect integrity and good faith, there are others who use them corruptly and dishonestly, to the pecuniary loss of their patients and the degradation of their profession.

The time has come for a reform, long looked for by all tenacious of professional honor, but now made necessary by public clamor. It can be accomplished, and at once, by a total abandonment of the practices upon which these charges of collusion are based. Let honorable members of the profession cease to write prescriptions upon the business cards of pharmacists. Let them, as soon as possible, vacate their offices adjacent to pharmacies. Let them abandon the use of private formulæ, cryptograms, known only to the initiated—and thus, like Cæsar's wife, be above suspicion.

To those who have already sunk so low as to have received commissions on prescriptions, if it is possible to reform that which must be already essentially deformed, we say, abandon either the profession or the dishonest practices which disgrace it.

The Society of Physicians and Surgeons, at its meeting August 11th, passed a series of resolutions condemning in the strongest terms the practices indicated above. Moreover, the resolutions were passed unanimously, and although there were but eighteen members present, these eighteen have placed themselves on record in their support. But this alone is not sufficient. The resolutions must be enforced. Let the Society look to this, and, having accomplished it, its whole duty to the profession and the public will have been fulfilled.

It remains now for the College of Pharmacy to consider their side of this question. If they can be induced—and we are assured by competent authority that they can—to pass, and enforce, resolutions comprehending the same objects, it will then be easy to crush out the evil between the two co-operating forces. H.

### *"Abortion Not a Crime."*

On the thirty-first day of July the notorious "Dr." Earll was convicted before the Criminal Court of Cook County of the murder of Rosetta Jackson, by abortion—the murder of the unborn child was entirely overlooked—and was sentenced by the jury to confinement for one year in the Penitentiary. The astonishment of all, and the indignation of many, who have heard or read the evidence in this case, at the utter disproportion of the punishment to the offense, will cease when the real reason shall have been ascertained. Here it is: Four of the jurors, convinced by the evidence that Earll had committed the abortion, were unwilling to convict, because they did not regard the act as criminal, considering that any one who did not desire offspring had a perfect right to destroy them in utero.

Here we have, in brief, the necessary result of the false teaching to which society has been subjected, both in physiology and morality—false teachings which have become a part of the moral character of the greater portion of society, under the sanction of mischievous laws, their inevitable outgrowth.

When the Civil Code shall have been reconstructed upon the basis of true physiology; when society shall have been taught that no arbitrary limit can be assigned as the initial point of a human life; when men, and, more especially, women, shall be convinced that a fœtus in utero, from its conception to its birth, is, potentially, as much a human being as the mother who bears it in her womb; when the truth which has been enunciated by medical jurists for a generation, that the destruction of the fœtus in utero, at whatever period of its existence, is murder, shall have been thoroughly understood; then will jurymen be made to know that the destruction of unborn children is not simply a matter of conscience, in which no one is concerned except the perpetrators, but that abortion is murder, planned by the woman, aided and abetted usually by some man, and perpetrated by some hired assassin dubbed "Dr.;" it may be in a Halsted street den by an "Earll," with his oil of tansy and his home-made hook; it may be by some one much better known, who earns his fifty dollars of blood-money in his office on a fashionable avenue, with an instrument much safer, an elastic bougie. We are told that there is no remedy for the evil; that it is simply an outgrowth of the innate depravity of human nature; that if one will not, another will, do these deeds of darkness and of blood; that women will produce abortion upon their own persons.

Moreover, we hear much of justifiable abortion to save the life of a pregnant woman; and we read in a cotemporary journal of recent date, of one eminent physician who performed five of these "justifiable" abortions which did not save the lives of the pregnant women, but destroyed them in every case; and we mentally thank God that we have not that load on our conscience, which all the honors of professional eminence can never counterbalance. If the justification of abortion rests upon the conscience and the judgment of any one man, why not upon that of any other man? What right has one physician to decide a question which another may not assume? If abortion is justifiable to save the life of a woman, why is it not equally so to save her reputation, which to many is dearer than life, whose loss has driven many to destroy life by suicide?

"*Falsus in uno, falsus in omnibus*"—that which is wrong, is so essentially, and by no sophistry, under no circumstances, can be made right.

There is a remedy for this great evil, a mode of preventing this terrible massacre of the innocents which is already making itself felt in essential ethnological modifications. Let physicians cease to throw the mantle of professional confidence around applications for the production of abortions. Let these be regarded, as they undoubtedly are, as suggestions to the commission of felony, and let them be treated as would be a suggestion to commit robbery, forgery, or perjury. Let information, under oath, be laid before the proper authorities that the crime of abortion is meditated, and we will see it rapidly diminish, and perhaps cease altogether.

***Chicago Medico-Historical Society.***

We have been favored by a professional friend with a glance at the proof sheet of what purports to be a corrected list of reputable and honorable members of the medical profession in Chicago, to be published under the sanction of this Medico-Historical Society. Its perusal suggested mingled feelings of amusement, indignation and regret: amusement at the utter incongruity of the resolution which precedes the list, and the list which follows it,—a resolution which deprecates abuses, practiced notoriously by many whose names are appended—indignation that this should go forth to the world as a corrected list of the honorable members of our profession in this city—and regret that gentlemen of intelligence, of integrity, of unblemished professional honor, should allow themselves to be used for such base purposes. We predicted in our June No. difficulties which must grow out of an institution so carelessly organized, but are ourselves surprised at the rapidity of their realization, and not less at their magnitude.

Gentlemen, there is one mode of escape from the perplexities which surround you. Dissolve, and reorganize, before that list shall have gone forth to the world as a fair exponent of the character and practices of your profession in this city. Is there no moral force in the profession? Shall it be said that two individuals dared to do what seventy-five dare not,—omit from their list men notoriously unprofessional, to use no stronger language?

Dissolve, before you demoralize the whole profession! H.

***Consistency (?).***

To receive into your private office for medical instruction pupils to whom you refuse admission into your medical society. For example, compare the resolutions of the Chicago Society of Physicians and Surgeons, passed by a large majority at its meeting, April 13, 1874, and the subsequent course of certain gentlemen who voted for that same resolution, and for further particulars "see small *bills.*" Gentlemen, remember the old Scotch proverb: "Betwixt twa stools the doup fa's doun,"—"Who serves two masters, must lie to one of them."

---

***Publishers' Notice.***

The Editors have crowded us with matter for the present No. to such an extent, that we have been compelled to print much of it "solid" instead of "leaded" as usual, for which we ask the indulgence of those of our readers who are compelled to wear spectacles. W. B. K., C. & Co.

---

***Errata.***

On page 417, July No. of the JOURNAL, in the foot-note, the word "depuration" should be *separation.*

On page 420, in the 18th line from the bottom, the word "labial" should be *lateral.*

---

***Cholera.***

OFFICE OF THE SUPERVISING SURGEON,
U. S. MARINE-HOSPITAL SERVICE,
*Treasury Department, August, 1874.*

DOCTOR:

The Supervising Surgeon of the United States Marine-Hospital Service having been designated by joint resolution of the Forty-third Congress, approved March 25, 1874, in connection with a medical officer of the Army, "to confer with the health authorities and resident physicians of such towns [as were visited by the Cholera Epidemic of 1873,] and to collect, so far as possible, all facts of importance with regard to such epidemic,"—for the purpose of making a report of the same to the President of the United States, to be submitted to Congress,—I have the honor respectfully to solicit a detail of the facts which came under your observation concerning the propagation and spread of the disease during that year.

The following memorandum embraces, substantially, the points upon which information is desired:

1. Name, sex, and age of patient.
2. Residence of patient—town, street, and number.
3. Day and hour of attack.
4. Premonitory symptoms, their nature and duration.
5. Progress of the disease: *a.* Day and hour of beginning of rice-water discharges; *b.* Day and hour of beginning of cramps; *c.* Day and hour of beginning of collapse; *d.* Period and extent of suspension of renal function; *e.* Nature of treatment and result; *f.* Day and hour when convalescence began; *g.* Day and hour when death occurred; *h.* Post-mortem appearances in detail.
6. Story of house occupied, and height of floor from ground.
7. Sanitary condition of house and enclosure: *i.* As to cleanliness of rooms—clean, neglected, filthy; *k.* As to ventilation and light—good, defective, bad; *l.* As to drainage of house—good, obstructed, absent; *m.* As to drainage of ground—good, obstructed, absent; *n.* As to location and condition of privies or water-closets, connection with street-sewer, mode of flushing, of ventilation of soil-pipe, disinfection, etc.; *o.* As to surface water, garbage, or filth about the premises.
8. Source and quality of water supply. If from a well or cistern, proximity of privy, sewer, or drain thereto, and chance of pollution.
9. General topography of localities in a given town where cholera prevailed.
10. Character of soil.
11. Character of drainage.
12. Occupation and habits of patient, and whether a resident of house where attacked for two weeks or over.
13. The facts in any case where the patient was attacked within two weeks after removing from an infected district into one previously free from the disease, specifying the respective districts and character of exposure.
14. The sequence of cases where more than one was attended, with their relations to each other, and to the cases of other physicians, with names of such physicians.
15. The means and agents used by the physician, by the family, and by the municipal authorities, to prevent the spread of the disease, and the result of such preventive measures.
16. Public measures taken to prevent the introduction of the disease into your community, with the result.
17. Temperature, rain-fall, and prevailing winds, for so long a period as practicable prior to appearance of cholera, and also during its continuance.
18. Dates of first and last cases of cholera in the locality in 1873—total number of cases, and mortality.
19. Connection, if any, between first cases in 1873, and the localities of the disease in the immediately preceding epidemic.
20. Names of cities, towns and villages known to you where cholera occurred during 1873, with any facts relating to the introduction of the disease to such, and the address of some respectable practitioner residing in each of the places named.

Contributions to this investigation, by answers to the foregoing—or to so much thereof as is practicable—will be fully acknowledged in the official report, the value of which, it is hardly necessary to say, will largely depend upon the co-operation of the profession thus sought.

Copies of any reports or papers which you may have already prepared on the subject, or of those prepared by others and annotated or emended by yourself, will also be of service, and may be forwarded, to be returned if desired.

I am, Doctor, very respectfully,

JNO. M. WOODWORTH,
*Supervising Surgeon.*

